# Longitudinal association between frequency of Internet use and incident disability among community-dwelling older people during the COVID-19 pandemic

**DOI:** 10.1265/ehpm.23-00207

**Published:** 2024-03-06

**Authors:** Kimiko Tomioka, Midori Shima, Keigo Saeki

**Affiliations:** Nara Prefectural Health Research Center, Nara Medical University, Kashihara, Nara, Japan

**Keywords:** Social distancing, COVID-19, Disability, Internet use, Prospective study, Older people, Japan

## Abstract

**Background:**

There is limited evidence of a protective effect of Internet use for incident disability (ID) during the COVID-19 pandemic. We investigated the association between frequency of Internet use (FIU) and ID among community-dwelling older people.

**Methods:**

We used longitudinal data from the 2019 and 2022 surveys, including 7,913 residents aged ≥65 without disability at baseline. ID was defined as a new public long-term care insurance certification. FIU at baseline was categorized into daily, weekly, monthly, yearly, and non-users. Changes in FIU before and during the COVID-19 pandemic were categorized into continuing frequent (i.e., daily or weekly), continuing moderate (i.e., monthly or yearly), increase in frequency, from non-users to users, decrease in frequency, from users to non-users, and continuing non-users. Covariates included age, gender, education, perceived economic situation, family structure, body mass index, chronic medical conditions, dietary variety, working status, walking time, and cognitive functioning. Multivariable Poisson regression models were used to estimate adjusted cumulative incidence ratio (aCIR) and 95% confidence interval (CI) for ID.

**Results:**

During the 3-year follow-up, 132 of 4,453 people aged 65–74, 595 of 3,460 people aged ≥75, 287 of 3,660 men, and 440 of 4,253 women developed ID. For FIU at baseline, among people aged ≥75 or men, there was a dose-response relationship between more frequent Internet use at baseline and a lower risk of ID (*P*-trend was 0.005 in people aged ≥75, and <0.001 in men). Compared to non-users, daily users had a significantly lower risk of ID [aCIR (95% CI) = 0.69 (0.53–0.90) in people aged ≥75, and 0.49 (0.34–0.70) in men]. For changes in FIU, “continuing frequent” and “from non-users to users” had a lower risk of ID than continuing non-users. After stratified analyses, “continuing frequent” remained a significant association in people aged ≥75 or in men, while “from non-users to users” had a significant association in those with daily walking time <30 minutes.

**Conclusions:**

Although FIU may act as a marker of disability, or indicate individual adaptability, our findings suggest that Internet use may be a potential preventive measure against ID in community-dwelling older people when social distancing is required.

**Supplementary information:**

The online version contains supplementary material available at https://doi.org/10.1265/ehpm.23-00207.

## Background

In countries, including Japan, where the aging of the population is progressing rapidly, preventing older people from requiring long-term care is an urgent issue to address, in order to ensure the sustainability of the social security system. Numerous studies have reported that social participation in old age is associated with a lower risk of developing disability [1–3]. This is because through social participation, older people can connect with other people, increase their physical activity, receive intellectual stimulation, and have a sense of purpose in life, which helps prevent deterioration of mental and physical functions. However, social distancing was required as a countermeasure to the global pandemic of the coronavirus disease 2019 (COVID-19) [4]. Therefore, it is feared that the restriction of social participation due to social distancing increases the risk of incident disability in older people.

Internet use is expected to help prevent community-dwelling older people from developing disability as a substitute for social participation [5]. There are two reasons for this. First, using the Internet enables non-face-to-face interactions, which may have a favorable effect on mental health by preventing loneliness and social isolation [6–8]. Good mental health has been reported to be associated with a lower risk of incident disability [9]. Second, Internet use by older people encourages them to actively access information, heighten their intellectual curiosity, and engage in intellectual activities [6, 10]. Practicing intellectual activities prevents cognitive decline in older persons and leads to the maintenance of independent living [11].

According to a survey by the Ministry of Internal Affairs and Communications [12], the percentage of Internet usage (individuals) among Japanese people in 2022 is reported to be 84.9%. In terms of the percentage of Internet usage by age group, this exceeds 90% for each age group from 13 to 59, while it tends to decrease with increasing age for those over 60 (86.8% for people in their 60s, 65.5% for people in their 70s, and 33.2% for people over 80). The proportion of Internet users among older people is lower than that of other age groups. If this study shows a significant association between Internet use and prevention of incident disability, it will provide a scientific basis for encouraging Internet use among community-dwelling older people in Japan, which has become a super-aging society.

We formed the following research hypothesis: 1) In a situation where interpersonal interactions are restricted due to the COVID-19 pandemic, Internet use by older people would alleviate the negative effects on mental health and prevent a new certification of need for long-term care. In other words, there may be a dose-response relationship between more frequent Internet use at baseline and a lower risk of incident disability three years later; 2) Even if the frequency of Internet use is significantly inversely associated with incident disability, older adults who use the Internet frequently may already have high levels of physical and mental activity [13]. If Internet use has a causal role in the development of disability, those who did not use the Internet at baseline but started using the Internet during the COVID-19 pandemic would be protected from developing incident disability. That is, we should assess the risk of incident disability in people who switched from non-users to users relative to those who continued to be non-users of the Internet, taking into account the effect of physical and mental activity levels.

In this study, we aimed to evaluate the association between the frequency of Internet use and incident disability using data from before the COVID-19 pandemic (i.e., October 2019) and during the COVID-19 pandemic (i.e., October 2022) based on a prospective cohort study of community-dwelling older people.

## Methods

### Study area and participants

**Fig. 1:**
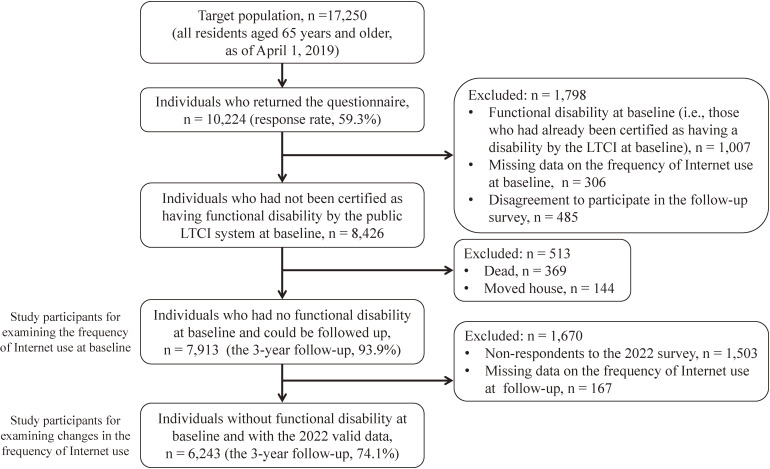
Flow chart of study participants. LTCI, Long-term Care Insurance.

The target area for this study was A City, Nara Prefecture, Japan. In this area, the aging rate, which is the proportion of people aged 65 and older in the total population, was 23.9% as of October 2022, which is lower than the national average (29.0%). Potential participants were all residents aged 65 and older as of April 1, 2019 (n = 17,250). In October 2019, A City distributed self-administered questionnaires to 17,250 people by mail, and received responses from 10,224 people (response rate: 59.3%). We followed 8,426 people who were not certified as requiring long-term care by the public long-term care insurance (LTCI) at the time of the 2019 survey (hereinafter referred to as the baseline), who agreed to participate in the follow-up survey, and had no missing data on the frequency of Internet use for 3 years (Fig. 1).

Of the 8,426 people, 7,913 people, excluding those who died or moved, were evaluated for the association between the frequency of Internet use at baseline and incident disability during the follow-up period. Of the 7,913 people, 6,243 people who had valid data on the follow-up survey in 2022 were examined for the association between the change in the frequency of Internet use and incident disability during the COVID-19 pandemic (from October 2019 to October 2022).

First, we compared the basic attributes of baseline survey respondents and non-respondents (Additional file 1). Non-respondents were more likely than respondents to be women, the youngest age group (65–69 years), the oldest age group (≥85 years), and people with functional disability. Next, we compared the baseline characteristics of three groups: those without follow-up, those without 2022 valid data, and the final analyzed participants (Additional file 2). Individuals who were lost to follow-up were more likely to be men, older, less educated, and have poor cognitive functioning, those without the 2022 valid data tended to be women, younger, of lower economic status, and had paid work, and many of the final analyzed participants were between the ages of 70 and 74, and had a high education. Finally, of the 9,838 people who answered on their frequency of Internet use at baseline, we assessed the frequency of Internet use of people with and without functional disability at baseline (Additional file 3). Among both age groups, there was a significant dose-response relationship between less frequent Internet use and higher prevalence of functional disability at baseline (*P* for trend < 0.001, Mantel-extension method). Characteristics of study participants are shown in Additional file 4.

### Assessment of outcome (incident disability)

Incident disability was determined based on data provided by A City regarding the status of certification under the public LTCI. In Japan, older people requiring nursing care can receive nursing care services based on the public LTCI [14]. The determination of whether or not an older person requires nursing care, in other words certification of long-term care need, is made by a committee for certification of need established in the municipality that is the insurer. Certification of long-term care need is based on nationally unified methods and standards [14, 15]. Specifically, investigators from the municipality conduct a “certification survey” with the older person and the family to assess the older person’s mental and physical state. The “certification survey” examines physical functioning, cognitive functioning, mental and behavioral disorders, social adjustment, and medical care received in the past 14 days. The committee for certification of need is composed of academic experts in health, medical care, and welfare, and assesses whether long-term care is required based on the results of the certification survey and the written opinion of the attending physician [15]. A previous study has shown that the status of certification under the public LTCI in Japan has a strong correlation with the Barthel index, which is often used as an assessment indicator for basic activities of daily living [16]. In this study, those who had not received certification of long-term care need at baseline were followed up for 3 years, and those who received certification of long-term care need at follow-up were defined as people who newly received certification of long-term care need (i.e., people with incident disability).

### Assessment of the independent variable (the frequency of Internet use)

The frequency of Internet use was measured using the question “How often do you use the Internet?” in both the baseline (October 2019) and follow-up (October 2022) surveys. Participants could answer “almost every day,” “several times a week,” “several times a month,” “several times a year,” or “none.” Regarding the change in the frequency of Internet use, first, we re-classified participants as frequent if they answered “almost every day” or “several times a week,” as moderate if they answered “several times a month” or “several times a year,” and as non-users if they answered “none.” Next, based on the re-classified frequency of Internet use in both the baseline and follow-up surveys, participants were divided into seven groups: continuing frequent, continuing moderate, increase in frequency, from non-users to users, decrease in frequency, from users to non-users, or continuing non-users.

### Covariates

Referring to previous studies [10, 11, 17–21], we adjusted for age, gender, family structure, perceived economic situation, education, chronic medical conditions, body mass index, dietary variety, working status, walking time, and cognitive functioning.

Family structure was categorized as living alone, living with only one’s spouse, living with a person other than a spouse, or living with three or more persons. Perceived economic situation was categorized as well off, normal, or poor. Education (years of schooling) was categorized as <10, 10–12, or >12 years. Chronic medical conditions included hypertension, diabetes, stroke, heart disease, and cancer. The number of chronic medical conditions undergoing treatment was categorized as none, one, or 2 or more. Body mass index (kg/m^2^) was categorized as thin (<18.5), normal (18.5–24.9), or overweight (25.0 or over). Dietary variety was evaluated using the Dietary Variety Score (DVS: score range 0–10) [20], and the lower tertile of DVS (DVS of 0–3) was defined as having low dietary variety. Working status was measured using the question “Are you currently in paid employment?” and participants were grouped by the presence or absence of paid work at baseline. Regarding walking time, previous studies in Japan have reported that daily physical activity levels in community-dwelling older people are associated with walking time [22, 23]. Participants were asked about walking time per day, and classified into the following three groups: ≥60 minutes, 30–59 minutes, or <30 minutes. Regarding cognitive functioning, previous studies have pointed out that cognitive decline can pose a barrier to Internet use for older people [13, 24]. Using the Cognitive Performance Scale (CPS) [25], cognitive functioning was categorized as poor (CPS scores of 1–6) or intact (CPS score of 0).

Regarding the variables used as covariates, none had a variance inflation factor greater than 2.0, confirming that there were no problems with multicollinearity. For missing covariate values, we treated them as the missing group to account for responses containing missing values [26].

### Statistical analyses

We used the generalized estimating equations of the multivariable Poisson regression models and estimated the cumulative incidence ratio (CIR) and 95% confidence interval (CI) for incident disability. The independent variable was the frequency of Internet use at baseline or the change in the frequency of Internet use before and during the COVID-19 pandemic. It has been reported that among Japanese people aged 60 and older, the proportion of Internet users is higher in men than in women, and decreases with age [27]. Previous studies of community-dwelling older people in Japan reported that the risk of developing incident disability was higher in women than in men, and increased with age [3, 28]. Because the association between the frequency of Internet use and incident disability may differ by age and gender, we conducted stratified analyses by age or gender. Moreover, the frequency of Internet use can be affected by physical and mental activity levels [13, 24]. Therefore, we also performed additional stratified analyses by physical and mental activity levels (i.e., walking time or cognitive functioning). The results of the stratified analyses by age or gender are presented below, while the results of additional stratified analyses are presented in the Supplementary material (i.e., Additional file 5).

We used the IBM SPSS Statistics Ver. 27 for Windows (Armonk, New York, US) for statistical analyses, and a significant level was set at 0.05 (two-tailed test).

## Results

During the 3-year follow-up, 727 out of 7,913 participants analyzed for the frequency of Internet use at baseline developed incident disability (the 3-year cumulative incidence of disability: 9.2%), and 435 out of 6,243 participants analyzed for the change in the frequency of Internet use developed incident disability (the 3-year cumulative incidence of disability: 7.0%). Among 7,913 participants analyzed for frequency of Internet use at baseline, compared to 7,186 participants without incident disability, 727 participants with incident disability were more likely to be female, be older, live alone, have fewer years of education, have more chronic diseases, have more abnormal body mass index, walk less, have poorer cognitive functioning, and be unemployed. There were no significant differences between the two groups with respect to perceived economic situation and dietary variety. The same trends were observed in 6,243 participants analyzed for changes in the frequency of Internet use (comparison of 5,808 people without incident disability and 435 people with incident disability) (Table 1).

**Table 1:** Baseline characteristics by incident disability over 3 years

**Baseline characteristics**	**Frequency of Internet use at baseline (n = 7,913)**					**Change in the frequency of Internet use (n = 6,243)**				
	
	**Without incident disability (n = 7,186)**		**With incident disability (n = 727)**		***P***	**Without incident disability (n = 5,808)**		**With incident disability (n = 435)**		***P***
	**n**	**(%)**	**n**	**(%)**		**n**	**(%)**	**n**	**(%)**	
Gender: men	3,373	(46.9)	287	(39.5)	<0.001	2,745	(47.3)	175	(40.2)	0.005
Age: 75 and older	2,865	(39.9)	595	(81.8)	<0.001	2,342	(40.3)	350	(80.5)	<0.001
Family structure: living alone	863	(12.0)	136	(18.7)	<0.001	702	(12.1)	75	(17.2)	0.002
Perceived economic situation: poor	1,693	(23.6)	169	(23.2)	0.855	1,326	(22.8)	103	(23.7)	0.723
Education (years of schooling): <10	1,249	(17.4)	194	(26.7)	<0.001	971	(16.7)	118	(27.1)	<0.001
Chronic medical conditions: present	4,022	(56.0)	477	(65.6)	<0.001	3,271	(56.3)	295	(67.8)	<0.001
Body mass index: normal	5,034	(70.1)	441	(60.7)	<0.001	4,078	(70.2)	266	(61.1)	<0.001
Dietary variety: low	2,416	(33.6)	255	(35.1)	0.434	1,927	(33.2)	147	(33.8)	0.833
Working status: non-working	5,057	(70.4)	605	(83.2)	<0.001	4,139	(71.3)	372	(85.5)	<0.001
Walking time: <30 minutes per day	1,674	(23.3)	252	(34.7)	<0.001	1,327	(22.8)	139	(32.0)	<0.001
Cognitive functioning: poor	1,217	(16.9)	264	(36.3)	<0.001	956	(16.5)	156	(35.9)	<0.001

*P* value was based on the Chi-squared test.

Dietary variety was evaluated using the Dietary Variety Score.

Cognitive functioning was evaluated using the Cognitive Performance Scale.

Regarding the association of Internet use frequency at baseline with incident disability (Table 2), among all participants, there was a dose-response relationship in which the higher the frequency of Internet use at baseline, the lower the risk of incident disability (*P* for trend < 0.001). In addition, compared with participants who did not use the Internet at baseline, participants who used the Internet almost every day had a significantly lower incidence of disability (adjusted CIR = 0.67, 95% CI = 0.53–0.85). The results of stratified analyses by age (the middle of Table 2) showed that a dose-response relationship between Internet use frequency and incident disability was significant in people aged 75 and older (*P* for trend = 0.005) but not in those aged 65–74 (*P* for trend = 0.135), and a significant difference between daily users and non-users was observed in people aged 75 and older (adjusted CIR = 0.69, 95% CI = 0.53–0.90) but not in those aged 65–74 (adjusted CIR = 0.66, 95% CI = 0.41–1.06). After stratified analyses by gender (right side of Table 2), men had a significant association between more frequent Internet use and lower risk of incident disability (*P* for trend < 0.001) and a significant difference between non-users and daily users (adjusted CIR = 0.49, 95% CI = 0.34–0.70). In contrast, among women, the frequency of Internet use at baseline was not associated with incident disability.

**Table 2:** Associations of Internet use frequency at baseline with incident disability by age or gender

**Frequency of ** **Internet use ** **at baseline**	**All participants**		**Age**				**Gender**			
	
			**Aged 65–74 (young-old)**		**Aged ≥75 (old-old)**		**Men**		**Women**	
	**(n = 7,913)**		**(n = 4,453)**		**(n = 3,460)**		**(n = 3,660)**		**(n = 4,253)**	
	**N**	**CIR^a^ (95% CI)**	**N**	**CIR^a^ (95% CI)**	**N**	**CIR^a^ (95% CI)**	**N**	**CIR^b^ (95% CI)**	**N**	**CIR^b^ (95% CI)**
None	3,436	1.00	1,345	1.00	2,091	1.00	1,248	1.00	2,188	1.00
Several times a year	396	0.83 (0.61–1.12)	228	0.46 (0.17–1.26)	168	0.91 (0.67–1.26)	185	0.70 (0.42–1.16)	211	0.93 (0.64–1.36)
Several times a month	532	0.97 (0.72–1.30)	331	0.72 (0.35–1.48)	201	1.05 (0.76–1.45)	266	1.00 (0.65–1.53)	266	0.94 (0.62–1.42)
Several times a week	1,005	0.83 (0.65–1.07)	663	0.87 (0.53–1.43)	342	0.81 (0.61–1.08)	501	0.88 (0.62–1.24)	504	0.79 (0.55–1.14)
Almost every day	2,544	0.67 (0.53–0.85)*	1,886	0.66 (0.41–1.06)^†^	658	0.69 (0.53–0.90)*	1,460	0.49 (0.34–0.70)**	1,084	0.90 (0.68–1.21)
	*P* for trend < 0.001		*P* for trend = 0.135		*P* for trend = 0.005		*P* for trend < 0.001		*P* for trend = 0.277	

CI, confidence interval; CIR, cumulative incidence ratio. **P < 0.001, *P < 0.05, ^†^*P* < 0.10.

The generalized estimating equations of the multivariable Poisson regression models were used as a method of statistical analysis. Outcome was incident disability.

^a^Adjusted for covariates (i.e., age, gender, family structure, perceived economic situation, education, chronic medical conditions, body mass index, dietary variety, working status, walking time, and cognitive functioning). ^b^Adjusted for covariates excluding gender.

Regarding changes in Internet use frequency (Table 3), among all study participants, the “from non-users to users” group (adjusted CIR = 0.68, 95% CI = 0.47–0.97) and the “continuing frequent” group (adjusted CIR = 0.59, 95% CI = 0.44–0.80) had a significantly lower incidence of disability than continuing non-users. After stratified analyses by age or gender, a significant difference between “continuing frequent” and continuing non-users remained, while no significant difference was found between “from non-users to users” and “continuing non-users”.

**Table 3:** Changes in Internet use frequency before and during the COVID-19 pandemic by age or gender

**Changes in ** **Internet use frequency ** **before and during the ** **COVID-19 pandemic**	**All participants**		**Age**				**Gender**			
	
			**Aged 65–74 (young-old)**		**Aged ≥75 (old-old)**		**Men**		**Women**	
	**(n = 6,243)**		**(n = 3,551)**		**(n = 2,692)**		**(n = 2,920)**		**(n = 3,323)**	
	**N**	**CIR^a^ (95% CI)**	**N**	**CIR^a^ (95% CI)**	**N**	**CIR^a^ (95% CI)**	**N**	**CIR^b^ (95% CI)**	**N**	**CIR^b^ (95% CI)**
Continuing non-users	2,053	1.00	741	1.00	1,312	1.00	779	1.00	1,274	1.00
From users to non-users	635	1.22 (0.95–1.58)	355	1.13 (0.62–2.08)	280	1.22 (0.92–1.61)	298	1.45 (1.01–2.08)*	337	1.08 (0.74–1.57)
Decrease in frequency	208	1.05 (0.64–1.72)	140	0.92 (0.32–2.69)	68	1.08 (0.63–1.86)	101	0.46 (0.17–1.24)	107	1.67 (0.96–2.92)^†^
From non-users to users	573	0.68 (0.47–0.97)*	300	0.45 (0.18–1.11)^†^	273	0.73 (0.50–1.07)	184	0.76 (0.43–1.37)	389	0.65 (0.42–1.02)^†^
Increase in frequency	315	0.72 (0.42–1.25)	228	0.58 (0.21–1.59)	87	0.80 (0.43–1.51)	135	0.56 (0.21–1.49)	180	0.88 (0.46–1.67)
Continuing moderate	146	1.03 (0.55–1.93)	86	0.97 (0.27–3.47)	60	0.98 (0.47–2.07)	77	0.80 (0.30–2.10)	69	1.21 (0.51–2.87)
Continuing frequent	2,313	0.59 (0.44–0.80)**	1,701	0.55 (0.29–1.02)^†^	612	0.63 (0.44–0.89)*	1,346	0.46 (0.29–0.73)**	967	0.80 (0.54–1.19)

CI, confidence interval; CIR, cumulative incidence ratio; Frequent, weekly or more; Moderate, monthly or yearly. **P < 0.001, *P < 0.05, ^†^*P* < 0.10.

The generalized estimating equations of the multivariable Poisson regression models were used as a method of statistical analysis. Outcome was incident disability.

^a^Adjusted for covariates (i.e., age, gender, family structure, perceived economic situation, education, chronic medical conditions, body mass index, dietary variety, working status, walking time, and cognitive functioning). ^b^Adjusted for covariates excluding gender.

To address the effects of the physical and mental activity levels, we conducted additional stratified analyses by walking time or cognitive functioning for changes in Internet use frequency (Additional file 5). A significant difference between “from non-users to users” and “continuing non-users” was observed in the group with a walking time less than 30 minutes per day (adjusted CIR = 0.39, 95% CI = 0.17–0.89), suggesting that Internet use may have a protective effect on incident disability in community-dwelling older people with low levels of physical activity.

## Discussion

This study examined the association between the frequency of Internet use and incident disability, using the 2019 and 2022 surveys’ data from a prospective cohort study of community-dwelling older people. First, we found that older adults who used the Internet daily before the COVID-19 pandemic had a lower risk of developing disability during the COVID-19 pandemic, and that the risk of developing disability decreased as the frequency of Internet use increased. For changes in the frequency of Internet use before and during the COVID-19 pandemic, we clarified that the risk of developing disability decreased in those who continued to use it frequently. Second, people who switched from being non-users before the COVID-19 pandemic to users during the COVID-19 pandemic had a significantly lower risk of incident disability than continuing non-users. To the best of our knowledge, this study is the first to demonstrate that frequent Internet use before and during the COVID-19 pandemic can prevent community-dwelling older adults from developing disability during the COVID-19 pandemic.

Not only in Japan, but also worldwide, Internet users have increased during the COVID-19 pandemic, which raises concerns about the increased risk of Internet addiction and its adverse effects on social life and health [29–32]. However, in this study, there was no adverse effect on incident disability due to frequent Internet use by community-dwelling older adults: Older adults who used the Internet almost every day had a significantly lower risk of disability than those who did not use the Internet at baseline. Furthermore, even when older people continued to use the Internet during the COVID-19 pandemic, the moderate frequency group had no effect in preventing incident disability, and only the frequent group reduced the risk of disability onset. These results suggest that in order to prevent incident disability, community-dwelling older adults need to use the Internet frequently, not only during the spread of emerging infectious diseases, but also during normal times.

Regarding previous research on Internet use among older people, we were unable to find any studies that investigated the association with disability, but many studies examined the association with mental health and reported both positive and negative consequences of Internet use. For Internet use before the COVID-19 pandemic, a longitudinal study in the UK reported that older people with daily Internet use had better life satisfaction compared to those with infrequent Internet use, but the frequency of internet use was not associated with depression [27]. Cross-sectional studies found that frequent Internet use in older people was associated with poor quality of life (QOL) [17] and poor mental health [28]. For Internet use during the COVID-19 pandemic, an English cross-sectional study of individuals aged 55–75 found that more frequent Internet users had lower depression symptoms and higher QOL [18]. A German cross-sectional study found that frequent contact with friends and relatives via the Internet was associated with less loneliness, greater life satisfaction, and less depressive symptoms [33]. Previous studies have the following limitations: Many of them had a cross-sectional design [17, 18, 28, 33–37], and even longitudinal studies evaluated Internet use frequency before the COVID-19 pandemic [27], or had a short follow-up period of one year [21]. Although more research is needed to establish a link between the frequency of Internet use and mental health among older adults, frequent Internet use during the COVID-19 pandemic may have a positive impact on mental health among older adults. Because poor mental health status such as psychological distress [9] and decreased QOL [38] is a risk factor for incident disability in community-dwelling older adults, frequent Internet use may have prevented the deterioration in mental health due to social distancing and prevented the onset of disability.

Regarding the mechanism of the association between the frequency of Internet use and incident disability among older people, in addition to the forementioned mental health aspect, we have two possible explanations. First, a previous study from China reported that Internet use by older adults increased their exercise frequency, resulting in improved physical health status [39]. In Japan, during the COVID-19 pandemic, in order to prevent a decline in physical strength due to lockdown restrictions, academic societies and local governments have created exercise videos that older people can do at home and applications that allow older people to exercise while self-managing online, and have released them on the Internet [40]. Because older people using the Internet can access these videos and applications, they may have increased their exercise frequency, leading to the prevention of incident disability. Second, a systematic review of the association between sedentary behavior and health suggests that Internet use increases sedentary time, which may increase the risk of obesity and cardiovascular disease in middle-aged adults, whereas Internet use may have a positive impact on cognitive functioning in older adults [41]. Because cognitive decline is a risk factor for developing disability [42], it is possible that Internet use may prevent cognitive decline in community-dwelling older adults and lead to a decrease in the risk of developing disability. This explanation supports our observed association between the frequency of Internet use and incident disability in people aged 75 and older: Because the risk of cognitive decline increases with age [42], the effect of Internet use on maintaining cognitive functioning may be greater for the old-old than for the young-old.

Regarding whether Internet use by older adults has a causal role in the development of incident disability, it has been pointed out that physical and mental limitations hinder Internet use in daily life [13, 24]. Therefore, older people who do not use the Internet may be in poor physical and mental health, and the possibility cannot be denied that they are vulnerable to the development of incident disability. Although those with functional disability at baseline were excluded from the study participants, we assessed the proportion of those with functional disability at baseline by the frequency of Internet use (Additional file 3), which showed that people who already had functional disability did not use the Internet so frequently at baseline. Therefore, the frequency of Internet use may act as a marker of disability levels, but not as a predictor of incident disability. Moreover, recent studies have reported that community-dwelling older adults have adapted to lifestyle changes such as social distancing during the COVID-19 pandemic by using the Internet [42, 43]. Therefore, adaptability to social environment changes, rather than Internet use itself, may prevent incident disability during the COVID-19 pandemic. However, our results showed not only a dose-response relationship between more frequent Internet use at baseline and a lower risk of incident disability, but also a low risk of incident disability in people who switched from non-users to users, which would tend to support our research hypothesis. Therefore, although we should be mindful that the observed association may be due to reverse causality or adaptability to environment, our findings suggest that Internet use may be one of the countermeasures to prevent the development of disability in community-dwelling older people during the spread of emerging infectious diseases.

This study has strengths. First, our study conducted an analysis based on a longitudinal study using a self-administered questionnaire, and succeeded in evaluating the frequency of Internet use before and during the COVID-19 pandemic. Second, because we evaluated not the increase or decrease in the frequency but the degree of frequency as the change in the frequency of Internet use, we could clarify the importance of maintaining frequent Internet use. Third, the outcome, or incident disability, was an objective index with high validity.

This study has limitations. First, this study was a prospective cohort study, those who had a disability at baseline were excluded from the analysis, and covariates were carefully considered. However, the 3-year follow-up period was not long enough to completely rule out the possibility of reverse causality. In the future, longer-term follow-up studies are needed. Second, previous studies would suggest that the impact of Internet use on mental health should be examined in terms of the purpose of Internet use, time spent on the Internet, and Internet skills: Frequent Internet users or those who used the Internet for communication purposes received a beneficial impact on their mental health, but Internet use for health-related information searching was associated with poor mental health [18, 37]. Prevalence of depression was higher in older adults with longer time spent on the Internet, and lower in those with higher Internet skills and in those who used the WeChat function [36]. Future research should evaluate the associations of these factors with incident disability. Third, although this study adjusted for important confounding factors, such as socioeconomic status, lifestyle habits, and physical and mental activity levels, the possibility of unmeasured confounding factors cannot be ruled out. For example, social support [34] and social capital [35] are known to mediate the association between Internet use and self-rated health among older adults. The observed associations may weaken if social support and social capital were added to the covariates. Fourth, the possibility of misclassification cannot be denied because the assessment of the frequency of Internet use was based on self-report. Misclassification may be leading to a weakening of the association between Internet use and incident disability. Fifth, in this study, many people were excluded from our analyses. Because the excluded individuals were more likely to be older or have a lower socioeconomic status than the analyzed participants (Additional files 1–2), it is possible that older adults with a high risk of developing disability were selectively excluded from our study. Finally, according to a survey of 25,585 older people aged 65 and older who are independent in their daily activities in 64 municipalities nationwide, 60.8% of them used the Internet several times a month or more, and there are differences between cities and towns in terms of the percentage of Internet users, with the percentage being higher in urban areas [44]. Of the 7,913 participants in this study, 51.6% used the Internet several times a month or more, which was about 10.0% lower than in a previous study. The low proportion of older adults using the Internet in this study may reduce the generalizability of the results.

Despite these limitations, our findings have several implications. We suggest to policy makers that Internet use is a potential effective strategy to prevent incident disability in community-dwelling older adults. We also recommend that general practitioners advise older patients to actively use the Internet to prevent functional decline during the spread of emerging infectious diseases.

## Conclusions

Our findings suggest that frequent Internet use may prevent community-dwelling older people from developing disability during the spread of emerging infectious diseases. However, because this study had a short follow-up period and the possibility of reverse causality and individual adaptability, our results should be interpreted with caution and further research is required.

## Abbreviations

CI: confidence interval
CIR: cumulative incidence ratio
COVID-19: Coronavirus disease 2019
CPS: Cognitive Performance Scale
DVS: dietary variety score
LTCI: long-term care insurance
QOL: quality of life
VIF: variance inflation factor

## Supplementary information

**Additional file 1.** Basic attributes of respondents and non-respondents to the baseline survey. **Additional file 2.** Those who were lost to follow-up, those without 2022 valid data, and final analyzed participants. **Additional file 3.** Frequency of Internet use at baseline of those with and without functional disability at baseline. **Additional file 4.** Characteristics of study participants. **Additional file 5.** Associations of changes in Internet use frequency with incident disability; additional stratified analyses.

## Declarations

### Availability of data and materials

No data and materials are available.
