# Supplementary material for: Longitudinal association between frequency of Internet use and incident disability among community-dwelling older people during the COVID-19 pandemic
Source: Environ Health Prev Med. 2024 Mar 6;29:13. doi: 10.1265/ehpm.23-00207 (PMC10937244; doi:10.1265/ehpm.23-00207)
Supplement: Supplementary file 1 — Additional file 1. Basic attributes of respondents and non-respondents to the baseline survey. Additional file 2. Those who were lost to follow-up, those without 2022 valid data, and final analyzed participants. Additional file 3. Frequency of Internet use at baseline of those with and without functional disability at baseline. Additional file 4. Characteristics of study participants. Additional file 5. Associations of changes in Internet use frequency with incident disability; additional stratified analyses. [file ehpm-29-013-s001.docx]

**Additional file 1**. Basic attributes of respondents and non-respondents to the baseline survey

| Basic attributes | | Respondents | Non-respondents | *P*-value^a^ |
| --- | --- | --- | --- | --- |
|  |  | (n = 10,224) | (n = 7,026) |  |
|  |  | n (%) | n (%) |  |
| Gender | |  |  |  |
|  | Men | 4,688 (45.9) | 3,034 (43.2) | 0.001 |
|  | Women | 5,536 (54.1) | 3,992 (56.8) |  |
| Age (years) | |  |  |  |
|  | 65–69 | 2,344 (22.9) | 1,785 (25.4) | <0.001 |
|  | 70–74 | 2,886 (28.2) | 1,869 (26.6) |  |
|  | 75–79 | 2,479 (24.2) | 1,472 (21.0) |  |
|  | 80–84 | 1,474 (14.4) | 951 (13.5) |  |
|  | ≥85 | 1,041 (10.2) | 949 (13.5) |  |
| Functional disability at baseline survey^b^ | | | |  |
|  | Absent | 9,217 (90.2) | 5,802 (82.6) | <0.001 |
|  | Present | 1,007 (9.8) | 1,224 (17.4) |  |

^a^Chi-squared test.

^b^Functional disability was deﬁned as persons who had already been certified as having a disability by the long-term care insurance as of October 31, 2019.

**Additional file 2**. Those who were lost to follow-up, those without 2022 valid data,

and final analyzed participants

|  | Baseline  characteristics | Lost to  follow-up^a^  (n = 1,304) | People without  2022 valid data^b^  (n = 1,670) | Final analyzed  participants^c^  (n = 6,243) | *P*^d^ |
| --- | --- | --- | --- | --- | --- |
| Gender | |  |  |  | 0.002 |
|  | Men | 663 (50.8) | 740 (44.3) | 2,920 (46.8) |  |
|  | Women | 641 (49.2) | 930 (55.7) | 3,323 (53.2) |  |
| Age | |  |  |  | <0.001 |
|  | 65–69 | 251 (19.2) | 447 (26.8) | 1,542 (24.7) |  |
|  | 70–74 | 349 (26.8) | 455 (27.2) | 2,009 (32.2) |  |
|  | 75–79 | 333 (25.5) | 384 (23.0) | 1,584 (25.4) |  |
|  | 80–84 | 209 (16.0) | 231 (13.8) | 810 (13.0) |  |
|  | ≥85 | 162 (12.4) | 153 (9.2) | 298 (4.8) |  |
| Perceived economic situation | | |  |  | 0.001 |
|  | Well off | 183 (14.0) | 250 (15.0) | 955 (15.3) |  |
|  | Normal | 776 (59.5) | 958 (57.4) | 3,780 (60.5) |  |
|  | Poor | 311 (23.8) | 433 (25.9) | 1,429 (22.9) |  |
|  | Missing | 34 (2.6) | 29 (1.7) | 79 (1.3) |  |
| Educational attainment | |  |  |  | <0.001 |
|  | <10 years | 292 (22.4) | 354 (21.2) | 1,089 (17.4) |  |
|  | 10–12 years | 602 (46.2) | 772 (46.2) | 3,084 (49.4) |  |
|  | >12 years | 351 (26.9) | 498 (29.8) | 1,952 (31.3) |  |
|  | Missing | 59 (4.5) | 46 (2.8) | 118 (1.9) |  |
| Working status | |  |  |  | <0.001 |
|  | Non-working | 949 (72.8) | 1,151 (68.9) | 4,511 (72.3) |  |
|  | Currently working | 254 (19.5) | 447 (26.8) | 1,560 (25.0) |  |
|  | Missing | 101 (7.7) | 72 (4.3) | 172 (2.8) |  |
| Cognitive functioning | |  |  |  | <0.001 |
|  | Intact | 903 (69.2) | 1,263 (75.6) | 5,028 (80.5) |  |
|  | Poor | 342 (26.2) | 369 (22.1) | 1,112 (17.8) |  |
|  | Missing | 59 (4.5) | 38 (2.3) | 103 (1.6) |  |

Data are given as n (%). ^a^Included individuals who declined to participate in the follow-up survey (n = 485), died (n = 369), moved house (n = 144), and those with missing data on the frequency of Internet use at baseline (n = 306). ^b^Included non-respondents to the 2022 survey

(n = 1,503) and those with missing data on the frequency of Internet use at follow-up (n = 167). ^c^Analyzed participants for the change in the frequency of Internet use. ^d^Chi-squared test.

**Additional file 3**. Frequency of Internet use at baseline of those with and without functional disability at baseline^a^

| Internet use frequency  at baseline | All (n = 9,836) | | |  | Aged 65–74 (n = 5,093) | | |  | Aged ≥75 (n = 4,743) | | |
| --- | --- | --- | --- | --- | --- | --- | --- | --- | --- | --- | --- |
|  | Functional disability at baseline | | |  | Functional disability at baseline | | |  | Functional disability at baseline | | |
|  | Absent  (n) | Present  (n) | Prevalence  of disability |  | Absent  (n) | Present  (n) | Prevalence  of disability |  | Absent  (n) | Present  (n) | Prevalence  of disability |
| Almost every day | 2,839 | 52 | 1.8% |  | 2,094 | 17 | 0.8% |  | 745 | 35 | 4.5% |
| Several times a week | 1,116 | 46 | 4.0% |  | 721 | 15 | 2.0% |  | 395 | 31 | 7.3% |
| Several times a month | 601 | 32 | 5.1% |  | 366 | 11 | 2.9% |  | 235 | 21 | 8.2% |
| Several times a year | 455 | 42 | 8.5% |  | 259 | 11 | 4.1% |  | 196 | 31 | 13.7% |
| None | 3,900 | 753 | 16.2% |  | 1,514 | 85 | 5.3% |  | 2,386 | 668 | 21.9% |
| All | 8,911 | 925 | 9.4% |  | 4,954 | 139 | 2.7% |  | 3,957 | 786 | 16.6% |

^a^This analysis excluded those with missing data on the frequency of Internet use at baseline (n = 388). This number does not match the number of people in Figure 1 (n = 306), because people with both functional disability and missing data are counted as having functional disability.

**Additional file 4.** Characteristics of study participants

| Characteristics | | Frequency of Internet use  at baseline (n = 7,913) | | | |  | Change in the frequency of  Internet use (n = 6,243) | | | |
| --- | --- | --- | --- | --- | --- | --- | --- | --- | --- | --- |
|  |  |  | n | (%) |  |  |  | n | (%) |  |
| Age | |  | Ave 74.1 | (SD 5.94) |  |  |  | Ave 74.0 | (SD 5.75) |  |
|  | 65–69 |  | 1,989 | (25.1) |  |  |  | 1,542 | (24.7) |  |
|  | 70–74 |  | 2,464 | (31.1) |  |  |  | 2,009 | (32.2) |  |
|  | 75–79 |  | 1,968 | (24.9) |  |  |  | 1,584 | (25.4) |  |
|  | 80–84 |  | 1,041 | (13.2) |  |  |  | 810 | (13.0) |  |
|  | ≥85 |  | 451 | (5.7) |  |  |  | 298 | (4.8) |  |
| Gender | |  |  |  |  |  |  |  |  |  |
|  | Men |  | 3,660 | (46.3) |  |  |  | 2,920 | (46.8) |  |
|  | Women |  | 4,253 | (53.7) |  |  |  | 3,323 | (53.2) |  |
| Family structure | |  |  |  |  |  |  |  |  |  |
|  | Living alone |  | 999 | (12.6) |  |  |  | 777 | (12.4) |  |
|  | Living with only one’s spouse |  | 3,821 | (48.3) |  |  |  | 3,101 | (49.7) |  |
|  | Living with a person other than a spouse |  | 457 | (5.8) |  |  |  | 336 | (5.4) |  |
|  | Living with three or more persons |  | 2,553 | (32.3) |  |  |  | 1,973 | (31.6) |  |
|  | Missing |  | 83 | (1.0) |  |  |  | 56 | (0.9) |  |
| Perceived economic situation | |  |  |  |  |  |  |  |  |  |
|  | Well off |  | 1,205 | (15.2) |  |  |  | 955 | (15.3) |  |
|  | Normal |  | 4,738 | (59.9) |  |  |  | 3,780 | (60.5) |  |
|  | Poor |  | 1,862 | (23.5) |  |  |  | 1,429 | (22.9) |  |
|  | Missing |  | 108 | (1.4) |  |  |  | 79 | (1.3) |  |
| Education (years of schooling) | |  |  |  |  |  |  |  |  |  |
|  | <10 years |  | 1,443 | (18.2) |  |  |  | 1,089 | (17.4) |  |
|  | 10–12 years |  | 3,856 | (48.7) |  |  |  | 3,084 | (49.4) |  |
|  | >12 years |  | 2,450 | (31.0) |  |  |  | 1,952 | (31.3) |  |
|  | Missing |  | 164 | (2.1) |  |  |  | 118 | (1.9) |  |
| The number of chronic medical conditions undergoing treatment | | | | | | | | |  |  |
|  | None |  | 3,228 | (40.8) |  |  |  | 2,552 | (40.9) |  |
|  | One |  | 3,306 | (41.8) |  |  |  | 2,632 | (42.2) |  |
|  | Two or more |  | 1,193 | (15.1) |  |  |  | 934 | (15.0) |  |
|  | Missing |  | 186 | (2.4) |  |  |  | 125 | (2.0) |  |
| Body mass index (kg/m^2^) | |  |  |  |  |  |  |  |  |  |
|  | Normal (18.5–<25.0) |  | 5,475 | (69.2) |  |  |  | 4,344 | (69.6) |  |
|  | Thin (<18.5) |  | 506 | (6.4) |  |  |  | 382 | (6.1) |  |
|  | Overweight (≥25.0) |  | 1,678 | (21.2) |  |  |  | 1,342 | (21.5) |  |
|  | Missing |  | 254 | (3.2) |  |  |  | 175 | (2.8) |  |
| Low dietary variety (DVS) | |  |  |  |  |  |  |  |  |  |
|  | Absent (4–10) |  | 5,026 | (63.5) |  |  |  | 4,020 | (64.4) |  |
|  | Present (0–3) |  | 2,671 | (33.8) |  |  |  | 2,074 | (33.2) |  |
|  | Missing |  | 216 | (2.7) |  |  |  | 149 | (2.4) |  |

**Additional file 4.** Continued

| Characteristics | | Frequency of Internet use | | | |  | Change in the frequency of | | | |
| --- | --- | --- | --- | --- | --- | --- | --- | --- | --- | --- |
|  |  | at baseline (n = 7,913) | | | |  | Internet use (n = 6,243) | | | |
|  |  |  | n | (%) |  |  |  | n | (%) |  |
| Working status | |  |  |  |  |  |  |  |  |  |
|  | Non-working |  | 5,662 | (71.6) |  |  |  | 4,511 | (72.3) |  |
|  | Currently working |  | 2,007 | (25.4) |  |  |  | 1,560 | (25.0) |  |
|  | Missing |  | 244 | (3.1) |  |  |  | 172 | (2.8) |  |
| Walking time (minutes per day) | | | |  |  |  |  |  |  |  |
|  | ≥60 minutes |  | 2,560 | (32.4) |  |  |  | 2,058 | (33.0) |  |
|  | 30–59 minutes |  | 3,131 | (39.6) |  |  |  | 2,513 | (40.3) |  |
|  | <30 minutes |  | 1,926 | (24.3) |  |  |  | 1,466 | (23.5) |  |
|  | Missing |  | 296 | (3.7) |  |  |  | 206 | (3.3) |  |
| Cognitive functioning (CPS score) | | | |  |  |  |  |  |  |  |
|  | Intact (zero) |  | 6,291 | (79.5) |  |  |  | 5,028 | (80.5) |  |
|  | Poor (one or higher) |  | 1,481 | (18.7) |  |  |  | 1,112 | (17.8) |  |
|  | Missing |  | 141 | (1.8) |  |  |  | 103 | (1.6) |  |
| Incident disability | |  |  |  |  |  |  |  |  |  |
|  | None |  | 7,186 | (90.8) |  |  |  | 5,808 | (93.0) |  |
|  | Present |  | 727 | (9.2) |  |  |  | 435 | (7.0) |  |
| Frequency of Internet use at baseline | | | |  |  |  |  |  |  |  |
|  | None |  | 3,436 | (43.4) |  |  |  |  |  |  |
|  | Several times a year |  | 396 | (5.0) |  |  |  |  |  |  |
|  | Several times a month |  | 532 | (6.7) |  |  |  |  |  |  |
|  | Several times a week |  | 1,005 | (12.7) |  |  |  |  |  |  |
|  | Almost every day |  | 2,544 | (32.1) |  |  |  |  |  |  |
| Change in the frequency of Internet use | | | |  |  |  |  |  |  |  |
|  | Continuing non-users |  |  |  |  |  |  | 2,053 | (32.9) |  |
|  | From users to non-users |  |  |  |  |  |  | 635 | (10.2) |  |
|  | Decrease in frequency |  |  |  |  |  |  | 208 | (3.3) |  |
|  | From non-users to users |  |  |  |  |  |  | 573 | (9.2) |  |
|  | Increase in frequency |  |  |  |  |  |  | 315 | (5.0) |  |
|  | Continuing moderate |  |  |  |  |  |  | 146 | (2.3) |  |
|  | Continuing frequent |  |  |  |  |  |  | 2,313 | (37.0) |  |

Ave, average; CPS, Cognitive Performance Scale; DVS, Dietary Variety Score; Frequent, weekly or more; Moderate, monthly or yearly; SD, standard deviation

**Additional file 5.** Associations of changes in Internet use frequency with incident disability; additional stratified analyses

**A.** By walking time

|  | Walking time (minutes per day) | | | | | | | |
| --- | --- | --- | --- | --- | --- | --- | --- | --- |
|  | >60 min (n = 2,058) | |  | 30–59 min (n = 2,513) | |  | <30 min (n = 1,466) | |
|  | N | CIR^a^ (95% CI) |  | N | CIR^a^ (95% CI) |  | N | CIR^a^ (95% CI) |
| Continuing non-users | 622 | 1.00 |  | 783 | 1.00 |  | 559 | 1.00 |
| From users to non-users | 203 | 0.98 (0.43-2.26) |  | 259 | 1.31 (0.92-1.86) |  | 151 | 1.15 (0.71-1.88) |
| Decrease in frequency | 58 | 1.54 (0.41-5.83) |  | 89 | 0.95 (0.42-2.15) |  | 53 | 0.98 (0.42-2.28) |
| From non-users to users | 188 | 0.77 (0.32-1.86) |  | 217 | 0.92 (0.57-1.46) |  | 147 | 0.39 (0.17-0.89)^*^ |
| Increase in frequency | 99 | 0.29 (0.08-1.09) |  | 130 | 0.63 (0.27-1.49) |  | 81 | 0.86 (0.36-2.09) |
| Continuing moderate | 50 | 3.01 (1.18-7.68)^*^ |  | 56 | 0.23 (0.03-1.66) |  | 38 | 1.43 (0.59-3.45) |
| Continuing frequent | 838 | 1.27 (0.64-2.52) |  | 979 | 0.51 (0.32-0.80)^*^ |  | 437 | 0.58 (0.33-0.99)^*^ |

**B.** By cognitive functioning

|  | Cognitive functioning | | | | |
| --- | --- | --- | --- | --- | --- |
|  | Intact (n = 5,028) | |  | Poor (n = 1,112) | |
|  | N | CIR^b^ (95% CI) |  | N | CIR^b^ (95% CI) |
| Continuing non-users | 1,466 | 1.00 |  | 542 | 1.00 |
| From users to non-users | 509 | 1.35 (0.99-1.85)^†^ |  | 117 | 0.94 (0.58-1.51) |
| Decrease in frequency | 169 | 1.11 (0.59-2.08) |  | 35 | 0.97 (0.39-2.42) |
| From non-users to users | 472 | 0.76 (0.49-1.17) |  | 94 | 0.56 (0.30-1.07)^†^ |
| Increase in frequency | 255 | 0.78 (0.39-1.57) |  | 56 | 0.65 (0.27-1.58) |
| Continuing moderate | 118 | 1.43 (0.71-2.89) |  | 27 | 0.70 (0.21-2.36) |
| Continuing frequent | 2,039 | 0.65 (0.45-0.93)^*^ |  | 241 | 0.53 (0.30-0.95)^*^ |

CI, confidence interval; CIR, cumulative incidence ratio. ^*^*P* <0.05, ^†^*P* <0.10.

The generalized estimating equations of the multivariable Poisson regression models were used as a method of statistical analysis. Outcome was incident disability.

Covariates include age, gender, family structure, perceived economic situation, education, chronic medical conditions, body mass index, dietary variety, working status, walking time, and cognitive functioning.

^a^Adjusted for covariates excluding walking time. ^b^Adjusted for covariates excluding cognitive functioning.
